# Fracture of the Skull

**Published:** 1828-02

**Authors:** 


### FRACTURE OF THE SKULL.

### *Cases of Compound Fracture of the Skull, with Depression. With Observations by* B. Travers, F.R.S.

Case I.—October 20th, 1827, William Clark, æt. thirteen, was brought into St. Bartholomew's Hospital, at eight P.M. in a state of stupor from a severe blow with a staff flung from a height, which had produced a radiated fracture of the left parietal bone, with depression. His pulse was small and irregular; skin cold; breathing hurried; frequent contractions of the muscles of the face and right arm; pupils moderately dilated, contracting feebly to the light; and much writhing of the body while the head was examined. The scalp wounds were dilated and joined, and four portions of depressed bone removed. Another large portion not detached was raised to the surrounding level, and a space equal to about two inches of the dura mater lay exposed.

He lost a few ounces of blood from the arm on the same night and succeeding day, and his bowels were kept gently open by castor-oil. On the 22d, (third day,) his pulse was 140, and he was disposed to coma, with oppression of the chest, and complained of pain in the head. These symptoms yielded to a dozen leeches applied to his temples, and motions obtained by castor-oil and a clyster. On the 25th, (sixth day,) he was convalescent.

After some days he had a smart attack of diffused inflammation of the connecting cellular tissue of the scalp and occipito-frontalis muscle, with much soreness and tumefaction on the opposite side of the head, and accompanied with fever. Twenty leeches were applied, and afterwards a cold lotion. He took a solution of salts with Liq. Antimon. Tartar., and the part recovered without suppuration; since which his progress to health has been uninterrupted.

Case II.—November 10th, 1827, John Williams, aged thirteen, was admitted into St. Bartholomew's Hospital, about noon, with a severe injury of the head from the falling of a wall after a fire. He was perfectly rational, and had no symptom of mischief to the brain, but a feeble pulse of sixty, and a cold surface.

A lacerated wound of the scalp near the posterior extremity of the sagittal suture on the right side of the cranium was dilated, and discovered a fracture extending from the suture, near its junction with the lambdoidal, across the parietal bone. The sagittal suture was loosened, and, at some distance from the former transverse section, another, commencing at the suture, took a diagonal direction. The triangular portion of bone included was driven in, and so wedged as to require the removal of a small portion of contiguous bone for the application of the elevator. A free venous hemorrhage occurred, which was checked by applying a dossil of lint. He lost rapidly near a pint of blood, and it was apprehended that the sinus was wounded. In the evening he was found quiet. His pulse had risen, he was sensible, and not complaining of pain, but disposed to sleep. In the morning he was heavy to sleep, having passed the night in that state without restlessness. He was perfectly sensible when spoken to, but unwilling to be roused; his pulse feeble, and intermitting occasionally. He had passed urine, and rejected a dose of castor-oil.

At two o'clock, the same state, drowsy, but rational; some blood had issued from the wound. The bowels had not acted, and four grains of Calomel and a common clyster were directed. These procured copious relief from the bowels.

Between five and six he expired, with little, if any, change of symptoms.

On examination, the exposed dura mater was found discoloured, and also a small wound in that membrane connected with a vein of the pia mater near its entrance into the longitudinal sinus, from which the hemorrhage had proceeded. The veins of the corresponding space of the pia mater were loaded, so as to give it a thickened appearance. The cortical substance was marked with specks of extravasation opposite the injury. Otherwise the brain was natural, firm, and healthy.

Case III.—Novomber 10th, 1827, Stephen Briffet, æt. twelve, was brought in along with the former, having received a similar injury at the same time and place. He had been stunned, but was now recovered. The skin, however, was chilled, pulse small, and breathing slow. A flap wound of the scalp over the left parietal bone, near its upper and posterior angle, exposed a fracture; and, after a farther dilatation of the exposed integument, a central portion of the bone, of the diameter of an inch, was observed to be insulated, and driven down upon the brain. From this a fissure extended towards the sagittal suture. The depressed piece was raised by forceps, without the aid of the trephine or saw. In addition to bruises, there was also a lacerated wound of the eyelids of the left eye at the external angle of the orbit, which had detached them and penetrated the conjunctiva, so as to lay open the orbit on this side to the bottom. In the evening, the boy was as well as could be desired, faculties perfect, and skin warm, with a moderate and soft pulse.

On the third day, having passed a restless night, his appearance and manner became altered. He was very restless, tossing about constantly, and complained of a pain in his head. The pupils of the eyes were dilated. The bowels having been opened, and the stomach rejecting medicine, forty drops of laudanum were injected in a clyster, and retained. This procured him five hours of sound sleep; but he continued to get worse, and died on the night of the 16th.

*Examination.*—A boundary of adhesive matter, corresponding to the fractured and denuded bone, was seen upon the dura mater, after raising the vault of the cranium. This membrane was sound. On raising it, the general appearance of this hemisphere was paler than that of the opposite side; the vessels of the pia mater being in a state of emptiness. The cerebral substance, on the contrary, exhibited very numerous red specks on section, and the cineritious substance opposite the injury was preternaturally loaded with blood. The chambers and basis of the brain were natural, but its substance soft.

*Observations.*—In all these cases the symptoms were those of commotion. In the first case there was evidence of pressure also, and of its relief by the operation, in the state of the senses, the breathing, the pulse, the pupils, &c. as compared before and after the operation. But the recurrence of similar symptoms on the third day, as well as the character of those first displayed, does not admit of this being entirely referred to the displacement and pressure of the fractured bone: it was, like the greater number of these accidents, a mixed case.

Whilst Mr. Travers was hesitating to operate upon Williams, the second case, it was observed that a circular piece of bone was apparently missing; but, upon gently dropping the probe through the aperture, it struck upon the surface of the displaced bone, and the apprehension of consequences from its complete impaction beneath the cranium determined him to proceed. It was by this piece that the dura mater had been torn, and, as the event proved, there would have been less risk in allowing it to remain; for it is probable that the boy's death was accelerated, under the existing circumstances, by the free hemorrhage from the pia matral vein.

Both these lads, Williams and Briffet, had been stunned, and laboured under the effects of severe physical shock or concussion. They were passively intelligent, pale, cold, with a small and feeble circulation. There was nothing in the symptoms that pointed to the head as the seat of injury. Their state was undistinguishable from that which we see every day produced by injuries of parts remote from the brain. Nevertheless they were in process of gradual recovery from the effects of a direct concussion of the brain, which an hour before had deprived them of sense and motion. The boy Clark (No. 1,) had been stunned to insensibility, and remained stupid, tossing his arms about, and moaning when disturbed for examination. He also was pale and cold; his pulse feeble and intermittent; he had rejected the contents of his stomach; his breathing was oppressed, not stertorous; pupils dilated, but not motionless. The relief following the operation in his case was marked and immediate.

The mode of injury by which a concussion is inflicted must influence not only the degree, but the character of the mischief. The singleness and purity of the concussion, or its combination with compression, creates a marked distinction.

The nature of a concussion, as of a fracture, is probably determined by the more diffused or circumscribed operation of the force by which it has been inflicted. Clark (No. 1,) was struck with a rail, or piece of wood, flung from a loft. Williams and Briffet were buried under the ruins of a falling wall.

Modern experiments have shown the difference in the effects (upon the organs which maintain life) of an injury which wounds or divides, and one which crushes or breaks down the structure of the brain or spinal marrow ; between the wound or removal of a part, and the disorganization of the mass. It would appear that the effects of a severe concussion are entailed upon the injury from the moment of its infliction, although the immediate symptoms of disturbance sometimes recede, and an interval of comparative tranquillity succeeds to it. For where no lesion of the brain or its membranes, nor any inflammatory action, has existed, how otherwise can we explain the recurrence of symptoms similar in character to those which mark the first stage,—that is, of purely nervous symptoms, after a period of three or more days, and their speedily fatal termination,—but by supposing a deeper and more universal injury to the nervous mass than was at first apprehended. The extraordinary frequency and conspicuousness of the red points, upon section of the medullary substance of the brain, Mr. Travers noticed repeatedly, contrasted with the empty state of the vessels upon the membranes, in cases in which the symptoms of shock had prevailed, both consequent upon and unconnected with injury of the head. A congested state of the capillary atteries to so considerable a degree as to render many conspicuous, which in a healthy brain are invisible, is a phenomenon opposed to that actually presented in fatal determinations to the head, in which the veins of the membrane are loaded, that in this are pale and collapsed. This congestion of the cerebral circulation in the arterial capillaries, or at least colourless vessels, must be gradual, and unlike that which happens in the general circulation at the moment of death. It is probably an effect of the impaired tone of the nervous structure, but it must exercise in turn a most extended influence on the actions of that system, and the functions of life. This, and the tendency to serous effusion, commonly regarded as the signs of inflammation, are the most notable appearances met with after death from concussion. Dr. Wilson Philip, in his chapter on Serous Apoplexy, (Enquiry into the Laws of the Vital Functions, p. 311,) has some interesting observations tending to show the analogy in the symptoms of concussion and of serous apoplexy, as contradistinguished to those, likewise analogous, of sanguineous apoplexy and compression. The depleting treatment cannot be borne in the former, which the latter calls for. Inflammation is equally remote from both.

Mr. Travers makes this observation as a caution to his juniors in the profession, against the premature employment of the lancet in concussion,—that is, before the circulation has completely recovered itself, or rather before the commencement of an action decidedly inflammatory. It is curious to observe how blindly the notion has been acted upon, which has been so generally and energetically impressed. The quantity of blood which a man can afford to part with in a given number of hours, is commonly exemplified by reference to cases of inflammation of the brain. Now, concussion and inflammation have unfortunately been identified, and lives have as undoubtedly been lost in the former, as saved in the latter, by the lancet. The reporters of the cases speak of a cold and pallid surface, and a sunken pulse, and its further sinking under the lancet, and their fear to proceed, in the same paragraph. Day after day, however, it has been resumed, as if in obedience to an abstract principle, and it is stated as matter of regret that only four or six ounces, instead of eight or twelve, as ordered, could be obtained at the last bleeding.

In most cases of concussion, the following treatment will, Mr. Travers thinks, be found most suitable. To relieve congestion, and no more, leech bleedings, and lax, not fretted, bowels: he prefers calomel or castor-oil, as the case requires, and the domestic clyster;—cold lotion to the shaved scalp, blisters to the occiput or nape of the neck; stimulant enemata, as of turpentine and assafœtida, if there be stupor and drowsiness; and if vigilance, tremors, and delirium, with a stomach too irritable to retain medicine, opiate clysters. The effect of both these remedies is excellent in the states described.

Cases of mixed symptoms are more frequent than such as admit of no mistake. In their descriptions, authors have displayed a regard for more exact and positive distinctions than nature commonly exemplifies. To give a system-like simplicity and an oracular tone to their directions, they have introduced diagnostics which are seldom seen, and still seldomer to be relied on. Take concussion and compression for example. The most frequent case is that in which the symptoms of commotion and pressure are so far blended as to make it doubtful whether, if not depending upon, they are not at least maintained by the displaced portion of bone. In a consultation, one party decides that it would be most prudent to elevate the depressed piece immediately, because, when inflammation is set up, and the symptoms become urgent, he considers that the operation aggravates the injury, and quickens the fatal event. The other questions this fact, and prefers to take the chance of the symptoms subsiding by abstinence, depletion, &c. Now, which of these parties is right? Thus far the question was tried under the observation of Mr. Travers, many years ago, at Guy's Hospital. Two boys were admitted, patients of the same surgeon, with compound fracture of the skull and depression. Neither had any symptoms of compressed brain, and consequently no operation was practised. After some days one of them was attacked with symptoms of inflammation, and the depressed bone was elevated. The symptoms were not in any degree mitigated, and the boy died. In the other the operation, which had been hitherto postponed, was now performed, and he did well. He might, however, have done as well had he been let alone, no symptoms having occurred; and the narrative therefore only shows, what we were predisposed to believe, that, where inflammation of the membrane follows upon depression, the operation is of no avail. Inflammation of the dura mater is a consequence of depression, but, if the parts be undisturbed, it may be limited to the spot, and the membrane be preserved from suppurating and spoiling. Mr. Travers would consider the advantage of being beforehand with the inflammation,—the many facts to prove that depression is by no means an irrecoverable condition,—the probability that if it be so inconsiderable as not to produce symptoms in the commencement, it may be prevented from afterwards doing so,—as full and sufficient grounds for declining the operation in all cases in which no real symptoms of compression presented themselves. On the other hand, the symptoms of compression being present, the operation, in his judgment, should be resorted to, whether the scalp were wounded or not. Thus Mr. Travers would make the presence of symptoms of compression, *cæteris paribus*, the criterion by which to determine the fitness of the operation; and the more marked the symptoms, the more indispensable the operation. And when, as in the case supposed, the symptoms are of a mixed nature, (and they are never absolutely separable,) we must be guided according to the predominance of either, being in mind that, in proportion as pure concussion prevails, any operation is objectionable. (*Medical Gazette.*)

